# 

**Published:** 1884-02

**Authors:** 


					﻿Whole Number
CCQCLXXIX.
February, 1884.
Number 2,
Vol. XLVIII.
TEE ZE
CHICAGO
MEDICAL
Journals Examiner.
CHICAGO:
PUBLISHED BY THE CHICAGO MEDICAL PRESS ASSOCIATION'
188 Clark Street.
t3F“Read the Notice of this Journal among Advertisements
				

